# The impact of Taekwondo instructors’ nonverbal communication on student engagement in liberal arts education: evidence from Chinese universities

**DOI:** 10.3389/fpsyg.2025.1668054

**Published:** 2025-11-27

**Authors:** Long Cheng, Yimeng Gu

**Affiliations:** Henan University, Kaifeng, China

**Keywords:** general education Taekwondo, nonverbal communication, classroom engagement behavior, positive emotions, recommended intention

## Abstract

**Background:**

In embodied disciplines like Taekwondo, instructor nonverbal communication (NVC) is a core pedagogical tool, yet its systemic impact on student engagement is often under-researched or treated as a monolithic outcome. This study analyzes the impact of Taekwondo instructors’ NVC on student engagement, operationalizing it as a multilevel construct comprising emotional, cognitive, and behavioral dimensions.

**Methods:**

A survey was conducted with 520 students enrolled in general education Taekwondo courses at five universities in China. Using a purposive sampling strategy, the study mapped Positive Emotions (as Emotional Engagement), Classroom Satisfaction (as Cognitive Engagement), and Recommendation Intention (as Behavioral Engagement) to examine their relationship with four NVC dimensions (appearance, auditory, body, and spatial language).

**Results:**

The analysis revealed a sequential process of engagement. First, Emotional Engagement (Positive Emotions) was significantly predicted by foundational NVC cues: visual, auditory, and body language. Second, Cognitive Engagement (Classroom Satisfaction) was similarly predicted by appearance, body, and auditory language. Third, high-level Behavioral Engagement (Recommendation Intention) was predicted by all four components, uniquely including spatial language.

**Conclusion:**

This study contributes a novel, tripartite model for assessing NVC’s total impact in sport pedagogy. We propose a sequential model where NVC first builds foundational trust and value (emotional/cognitive) and then, specifically through spatial cues, fosters the sense of belonging necessary for behavioral advocacy. These findings provide crucial data for the revitalization and teacher training of liberal arts Taekwondo classrooms.

## 1 Introduction

With economic development and improved quality of life, sports participation in China has been steadily increasing (Duan et al., 2022). Among them, Taekwondo is more popular than other sports, and Taekwondo gyms can be easily found in most cities and towns across China. China officially embraced Taekwondo when the 103rd International Olympic Committee session in Paris, France, in 1994 recognized it as an official competition event. It can be said that Taekwondo became popular in China because it was selected as an official competition event for the National Games, which is even more popular than the Olympics in China.

According to China’s Xinhua News Agency, on January 22, 2019, the Chinese Ministry of Education included Taekwondo as a subject in the high school entrance examination. Taekwondo offers various advantages, such as character education benefits, physical fitness, patience, etiquette, stability, low cost, and popularity. Due to these policies, China’s interest in Taekwondo has been increasing daily. The increasing attention toward Taekwondo in China is due to its ease of learning for beginners compared to Chinese martial arts, which are difficult to master and require long training periods. Additionally, Taekwondo has positive effects on physical and mental exercise as well as stress relief. Therefore, compared to other traditional martial arts, Taekwondo is relatively popular in contemporary China. Given its popularity, many universities in China have introduced Taekwondo as a general education course to enhance the educational level of Chinese citizens. It meets societal demands.

Studies have shown that research on university Taekwondo courses reveals that these courses enhance students’ Taekwondo proficiency in terms of content and knowledge, while also motivating them to actively participate in physical exercise in the future. The study also emphasizes the importance of Taekwondo education, stating that it plays a positive role in enhancing personal cultivation, psychological literacy, and willpower. Furthermore, by participating in the course, students can gain personal interest satisfaction and expansion, improve their social skills and meet social needs, and experience diverse classroom interactions, which maximizes their intrinsic psychological motivation. Therefore, this study argues that Taekwondo, as an essential component of general physical education courses, is highly worthy of research.

While these courses are beneficial, their success is dependent on instruction. In an embodied art such as Taekwondo, an instructor’s nonverbal communication (NVC) is an integral instructional structure. It serves as the primary medium of “teacher immediacy, “which refers to the nonverbal behaviors (e.g., gestures, tone, and eye contact) that minimize psychological distance, which allows students’ to build rapport.

Although prior research addressing the NVC-performance link in physical education, which we find useful, nearly always has a paramount theoretical issue: it often depersonalizes student engagement as a unitary, simplistic outcome (e.g., “satisfaction” or “motivation”), entreating it as a unique, regard for reputation. This theoretical treatment ignores the academic complexity of engagement as a multidimensional construct. Recent pedagogical theory (Zandi et al., 2025; Sulis and Mercer, 2025), has convincingly articulated that engagement must be explored as a definition that contains multiple, distinct emotional, cognitive, and behavioral dimensions.

It is currently unknown how specific NVC cues (e.g., an instructor’s appearance vs. their use of space) uniquely impact these different levels of engagement. This study fills that critical theoretical gap.

In this study we will explore NVC effects on multilevel engagement operationalized as Emotional (Positive Emotions), Cognitive (Classroom Satisfaction), and Behavioral (Recommendation Intention). In sum, we aim for an engagement model that is more comprehensive by suggesting a sequential model, environment where NVC first serves to establish essential credibility (emotional/cognitive dimension), and subsequently through specifically designated cues, support high level advocacy (behavioral dimension) in order to revitalize liberal arts Taekwondo.

## 2 Research questions and goals

Most of the current studies of nonverbal communication in general Taekwondo classes emphasize the nonverbal aspects related to teacher attractiveness, teacher image, and student motivation to participate (Kim and Jang, 2020). However, there has not been much research strictly regarding nonverbal communication. This study takes the nonverbal communication competence of instructors in general Taekwondo courses offered at universities in China, and through empirical analysis, explores the relationship between the instructors’ nonverbal communication competence and classroom engagement behaviors of students, how they are related and influence one another.

The goal of this research is to explore the impact of Taekwondo instructors’ nonverbal communication-auditory language, body language, visual language, and spatial language-on students’ engagement behaviors (e.g., classroom satisfaction, positive emotions, and intention to recommend the class). In this exploration, the study will emphasize the nature of instructors’ nonverbal communication competence in real teaching environments and possible recommendations from findings published in the idiographic qualitative research message. Finally, this research intends to provide the framework for future research on nonverbal communication and Taekwondo courses.

## 3 Research significance

In terms of theory, and with the knowledge that there is limited systematic research on nonverbal communication—especially on the effects of teacher behaviors for student participation—in general Taekwondo coaching, this research engages in general Taekwondo courses at Chinese universities. It establishes how certain components of nonverbal communication (appearance language, body language, and vocal language) influence students’ classroom satisfaction, positive emotions, and intention to recommend the course, and contributes to the theoretical buildout of this area. It also extends the nonverbal communication theory applications by expanding the contexts from commerce to sport pedagogy. By undertaking this empirical testing, this study verifies how Taekwondo classrooms assess nonverbal communication, enhances the applicability of nonverbal communication theory in educational contexts, and offers new aspects for future studies.

From an application-focused perspective, this research provides educational opportunities to maximize learning and recommendations for instructors to self-manage their own instructional strategies.

In conclusion, this research offers both cross-discipline potential and the possibility for cultural application of nonverbal communication theory and practical principles, leveraging scientific knowledge to inform Taekwondo practice resulting in important academic and practical contributions.

## 4 Literature review

### 4.1 Nonverbal communication

In the context of physical education (PE) and sport instruction, nonverbal communication (NVC) can be most effectively examined through the pedagogical framework of teacher immediacy. Immediacy refers to communicative behaviors that reduce the perceived psychological distance between instructors and students (Mehrabian, 1971). Within PE settings—where teaching relies heavily on physical demonstration, modeling, and emotional attunement—nonverbal immediacy (e.g., smiling, eye contact, forward lean, and affirmative gestures) functions as a core instructional tool rather than merely an interpersonal skill (García-Fariña, 2025).

Extensive research within sport pedagogy and related educational fields has confirmed the crucial role of NVC in fostering student engagement and learning. Empirical studies have consistently shown that PE instructors’ nonverbal immediacy predicts a wide range of positive student outcomes, including increased motivation, affective learning (i.e., students’ positive attitudes toward PE), and even improved cognitive acquisition of motor skills (Pishghadam et al., 2019; Bum and Lee, 2016). In physically embodied disciplines such as Taekwondo, the instructor’s body serves simultaneously as a medium of instruction and a message of emotional support. Thus, the nonverbal cues conveyed—such as a confident tone, expressive gestures, and professional appearance—are fundamental to bridging the gap between physical demonstration and student engagement (Khuman, 2024).

While general communication theory defines NVC as the transmission of meaning through nonverbal symbols (Burgoon et al., 1990), this study adopts a contextualized pedagogical perspective. Drawing on PE-specific literature, we conceptualize instructors’ nonverbal communication competence across four observable dimensions: auditory language (tone, volume, clarity), body language (gesture, facial expression), visual language (appearance, attire), and spatial language (distance, eye contact). This multidimensional approach allows a more precise analysis of how Taekwondo instructors’ nonverbal behaviors influence students’ classroom engagement in Chinese universities.

### 4.2 Classroom engagement behavior

Classroom engagement behavior refers to students’ observable actions that demonstrate their active participation in the teaching and learning process. However, as recent pedagogical research increasingly emphasizes, focusing solely on observable behaviors is insufficient, as it overlooks the underlying cognitive and emotional processes that sustain meaningful participation (Zandi et al., 2025).

Contemporary engagement theory, particularly the multilevel framework advanced by Sulis and Mercer (2025) and Zandi et al. (2025), conceptualizes engagement as the simultaneous co-activation of three interrelated dimensions: behavioral (actions), cognitive (psychological investment), and emotional (affective reactions).

This holistic model is uniquely suited for the PE context, as embodied disciplines like Taekwondo require the inherent interconnection of all three. A student must execute a physical kick (behavioral), while understanding its application and form (cognitive), and channeling focus and spirit (emotional).

To operationalize this framework, this study maps our three dependent variables onto these dimensions as follows:

1. Emotional Engagement—a student’s affective connection to the class—is operationalized as the Positive Emotions variable. Items measuring feelings such as “happy, ““excited, “and “cheerful” directly assess students’ positive affective experiences during the Taekwondo course (Weber et al., 2016).
2. Cognitive Engagement—a student’s psychological investment and evaluative judgment—is operationalized as the Classroom Satisfaction variable. Items such as “academically accomplished” and “professionally helpful” require a reflective appraisal of the course’s value. This is a higher-order cognitive process, representing a student’s evaluative judgment of their learning and its utility (Pietarinen et al., 2014).
3. Behavioral Engagement—a student’s actions and future-oriented intentions—is operationalized as the Recommendation Intention variable. While classroom participation reflects immediate behavior, the willingness to recommend the course is a pivotal, future-oriented advocacy action. This serves as a robust indicator of sustained engagement and loyalty that goes beyond mere attendance.

By explicitly mapping these variables, this study provides a novel, tripartite model for assessing NVC’s total impact in sport pedagogy. This approach allows for a comprehensive examination of how instructors’ nonverbal communication (NVC) shapes not only students’ outward behaviors but also their internal cognitive evaluations and emotional experiences.

## 5 Research methods

### 5.1 Research target

The students in this study were enrolled in general courses in Taekwondo at five universities in the Henan, Hebei, Guangdong, Guangxi, and Guizhou Provinces of China. A purposive sampling strategy was employed to recruit participants. The researchers visited five universities, after receiving permission from Taekwondo instructors, approached students who were present in casual environments (e.g., lounges or waiting areas). A total of 550 questionnaires were distributed (targeting approximately 110 per university) to students who were enrolled in the general education Taekwondo courses and willing to participate. At the end of this process, 520 valid questionnaires remained for analysis and the demographics of the participants are reported in Table 1.

**Table 1:** General characteristics of the study participants.

Categorization	Elements	Number of people	Frequency (%)
Genders	Boys	158	30.4
	Girls	362	69.6
Grade	Grade1	279	53.7
	Grade 2	95	18.3
	Grade 3	63	12.1
	Grade 4	83	16.0
Corrections Course Selection Path	Recommended by friends	114	21.9
	School announcement	130	25.0
	Like taekwondo.	99	19.0
	Want to learn Taekwondo	177	34.0
	Total	520	100.0

### 5.2 Selection, modification and design of survey instruments

The selection of the dependent variables was deliberately guided by our multilevel engagement framework (see Section 4.2), which aligns with recent pedagogical theory (Zandi et al., 2025; Sulis and Mercer, 2025). This study’s aim was not to assess psychomotor learning (e.g., skill acquisition), but rather the psychological and pedagogical dimensions of student engagement. Consequently, we selected three well-established variables to serve as direct operationalizations of the three distinct facets of engagement: Positive Emotions was chosen to measure Emotional Engagement; Classroom Satisfaction was chosen to measure Cognitive Engagement, representing the student’s evaluative judgment of the course’s value; and Recommendation Intention was chosen as a robust, future-oriented proxy for Behavioral Engagement (i.e., advocacy and loyalty).

The survey instruments used in this study are as follows. First, based on the study by Sundaram and Webster (2000), questions related to the nonverbal communication of general Taekwondo instructors at Chinese universities were proposed. Scholars such as Puertas-Molero et al. (2022) categorized nonverbal communication into four sub-factors in their studies: body language, spatial language, visual language, and auditory language, with a total of 16 items, four for each sub-factor.

Secondly, the items measuring ‘classroom satisfaction’ in this study were developed from and drew on established student questionnaires, namely the U.S. National Survey of Student Engagement (NSSE), the UK’s National Student Survey (NSS), and Australia’s Course Experience Questionnaire (CEQ). The items regarding teaching satisfaction were compiled and synthesized from these instruments, and modified and finalized for the China national context and participants in this study. In the current study classroom satisfaction is defined as unidimensional, consisting of 6 items.

Third, based on the study by Chamberlain and Broderick (2007), items related to positive emotions in participant behavior were examined. It consists of six questions used in the studies by Park et al. (2015).

The Recommendation Intention in Participation Behavior question was developed by scholars such as Babakus and Boller (1992) and Cronin Jr and Taylor (1992), as well as many other scholars, who used the scale in their articles. This article makes its own adjustments based on the characteristics and use of the study population.

At the same time, the survey questions selected through the review of previous studies were confirmed and data collection was conducted through the following procedures. First, the selected survey instruments were modified and supplemented to fit the purpose of this study and were presented to five professors specializing in Taekwondo and sports management, who then reviewed the validity and applicability of the questions. Second, since the questionnaire needed to be translated from a foreign language into Chinese for the survey, experts in English, Korean, and Chinese were invited to carry out the translation work. Additionally, to ensure the effectiveness of this process, five experts proficient in English, Chinese, and Korean participated in the translation. In this process, the experts were asked to translate the foreign-language questionnaire into Chinese for review, and then translate it back into the original language for verification. We attempted to minimize translation errors in the questionnaire through the above cross-verification method.

After completing the above work, the validity and applicability of the actual survey content were confirmed, and the final questionnaire was finalized through review. A pilot survey was conducted with 100 Chinese students. After collecting the pilot survey data, the validity of the questionnaire was confirmed through exploratory factor analysis. The reliability of the questionnaire was analyzed through reliability verification to ensure its validity and reliability. A five-point Likert scale was used, covering all questions except demographic characteristics, ranging from “strongly disagree” (1 point) to “strongly agree” (5 points).

### 5.3 Validity and reliability of survey instruments

To ensure the validity and reliability of the survey questions, we conducted a confirmatory factor analysis (CFA) on teachers’ nonverbal communication and participant behaviors (classroom satisfaction, positive emotions, and recommendation intention). The analysis results, as shown in Table 2, indicate that the model fit indices are *X*^2^ = 1015.583 (df = 413, *p* = 0.000), *X*^2^/df = 2.459, CFI = 0.952, TLI = 0.946, SRMR = 0.041, RMR = 0.022, and RMSEA = 0.053. Based on standard values such as CFI (≥0.9) and RMSEA (≤0.08), the model was determined to be appropriate. Next, to analyze convergent validity, factor loadings and measurement error values were examined, and based on these, composite reliability (CR) and average variance extracted (AVE) values were calculated. Considering these factors, the factor loadings ranged from a minimum to a maximum of 0.927, CR values ranged from a minimum to a maximum of 0.975, and AVE values ranged from 0.648 to 0.865. Therefore, factor loadings (≥0.5), CR values (≥0.7), and AVE values (≥0.5) all met the standards proposed in previous studies (Hair et al., 2006). Additionally, the results of the reliability analysis for each variable were as follows: auditory language is 0.926, body language is 0.841, visual language is 0.888, spatial language is 0.809; classroom satisfaction is 0.904, positive emotions is 0.904, and recommendation intention is 0.948. Therefore, analyzing the reliability values of all survey items using Cronbach’s alpha showed that all variables exceeded 0.7, meeting the reliability standard (≥0.7) proposed by Nunnally and Bernstein (1994).

**Table 2:** Confirmatory factor analysis and reliability analysis of all problems.

Variant	Problems	Factor loading values	Measured error value	CR (AVE)	Reliability
Auditory language	Moderate pace of speech	0.904	0.093	0.955(0.843)	0.926
	Moderate voice	0.899	0.127		
	Explain in a confident voice	0.844	0.189		
	Accurate pronunciation	0.857	0.161		
Body language	Use gestures to help with understanding	0.796	0.235	0.880 (0.648)	0.841
	body toward me	0.799	0.248		
	Nod in response	0.688	0.456		
	Instruct with a kind expression	0.751	0.315		
Appearance language	Dress well	0.841	0.118	0.943 (0.807)	0.888
	Have an attractive appearance	0.831	0.164		
	Have a good impression	0.852	0.121		
	Have a suitable body	0.753	0.241		
Spatial language	Explain by making eye contact	0.726	0.244	0.896 (0.684)	0.809
	Describe in a comfortable position	0.754	0.191		
	Be courteous to students	0.783	0.160		
	Frequency of contact is appropriate	0.651	0.410		
Course Satisfaction	Courses made you feel satisfied with school life	0.862	0.134	0.950 (0.793)	0.904
	Courses made you feel academically accomplished	0.864	0.138		
	Courses were academically important	0.833	0.160		
	Courses were professionally helpful	0.749	0.231		
	Overall satisfaction with courses	0.743	0.196		
Positive emotions	Thinking about taekwondo class makes me happy	0.927	0.067	0.975 (0.793)	0.948
	Thinking about Taekwondo class makes me feel excited.	0.910	0.081		
	Thinking about Taekwondo class makes me feel relaxed.	0.879	0.100		
	Thinking about Taekwondo class makes me feel cheerful.	0.910	0.085		
	Thinking about Taekwondo class makes me feel agreeable	0.813	0.168		
	Thinking about Taekwondo class makes me feel satisfied.	0.758	0.202		
Recommended intent	Recommend this class to others	0.875	0.109	0.953 (0.837)	0.905
	Recommend this class to your school	0.889	0.096		
	Recommend this class to a friend	0.882	0.106		
	Recommend this class to the Internet	0.724	0.247		
*X*^2^ = 1015.583 (df = 413, *p* = 0.000) CFI = 0.952, TLI = 0.946, SRMR = 0.041, RMR = 0.022, RMSEA = 0.053					

### 5.4 Data processing methods

The data processing methods in this study utilized SPSS 23.0 and AMOS 23.0 for data analysis. First, a frequency analysis was conducted to examine the demographic characteristics of the study participants, and confirmatory factor analysis (CFA) along with reliability analysis was performed to assess the validity and reliability of the questions.

Finally, a correlation analysis was carried out to explore the relationships between variables. To further assess multicollinearity, variance inflation factors (VIFs) were calculated for the four NVC predictor variables. All VIFs were found to be well below the standard threshold of 5.0, confirming the correlation table’s finding that multicollinearity was not a confounding issue for the regression analyses.

Multiple regression analysis was conducted to examine the impact of teachers’ nonverbal communication on the three dimensions of engagement (classroom satisfaction, positive emotions, and recommendation intention). While a unified model using Structural Equation Modeling (SEM) is a valuable approach, separate multiple regression models were intentionally chosen for this study’s primary objective. This objective was to gain a clear and direct diagnostic understanding of how each NVC component independently predicts each distinct dimension of engagement. This exploratory approach allows for the clearest identification of the specific relationships (e.g., the unique role of spatial language) that inform the sequential model we propose in our Discussion (Section 7). As noted in our limitations (Section 7.4), testing this unified sequential model via SEM is the critical next step for future research.

## 6 Results of the study

### 6.1 The correlation analysis results of all variables

The correlation analysis results of all variables are shown in Table 3. The analysis results indicate that all variable correlation values are significant, ranging from 0.253 to 0.732, with no exceptionally high correlation values. Therefore, there is no issue of multicollinearity as suggested in the literature (Kline, 2005), since no correlation exceeds 0.85.

**Table 3:** The correlation analysis results of all variables.

Variant	1	2	3	4	5	6	7
Auditory language	1						
Body language	0.312**	1					
Appearance language	0.402**	0.529**	1				
Spatial language	0.253**	0.536**	0.585**	1			
Course satisfaction	0.430**	0.501**	0.533**	0.406**	1		
Positive emotions	0.424**	0.474**	0.530**	0.403**	0.732**	1	
Recommended intent	0.392**	0.441**	0.526**	0.443**	0.609**	0.652**	1

**p < 0.01.

### 6.2 The impact of teachers’ nonverbal communication on classroom satisfaction

In the nonverbal communication of general Taekwondo instructors at Chinese universities, visual language (*β* = 0.279), body language (*β* = 0.258), and auditory language (*β* = 0.225) have a significant impact on classroom satisfaction. The study found that spatial language (*β* = 0.048) does not have a significant impact on classroom satisfaction (see Table 4). Additionally, the results of multiple regression analysis indicate that the explanatory power of the model is 38.9% (*R*^2^ = 0.394, △*R*^2^ = 0.389), with a significant model result (*F* = 83.690, *p* < 0.001).

**Table 4:** The impact of nonverbal communication on classroom satisfaction.

Variant	B	SE	*β*	*t*	*p*
Auditory language	0.196	0.033	0.225	5.966***	0.000
Body language	0.223	0.037	0.258	5.973***	0.000
Appearance language	0.282	0.047	0.279	6.011***	0.000
Spatial language	0.051	0.048	0.048	1.066	0.287
*R*^2^ = 0.394		△*R*^2^ = 0.389		*F* = 83.690, *p*<0.000	

***p < 0.001,**p < 0.01,*p < 0.05.

### 6.3 The impact of teachers’ nonverbal communication on positive emotions

In the nonverbal communication of general Taekwondo instructors at Chinese universities, visual language (*β* = 0.288), auditory language (*β* = 0.225), and body language (*β* = 0.219) have a significant impact on positive emotions in participation behavior, while spatial language (*β* = 0.060) does not have a significant impact (see Table 5). Additionally, the results of multiple regression analysis indicate that the explanatory power of the model is 37.1% (*R*^2^ = 0.376, △*R*^2^ = 0.371), showing a significant model (*F* = 77.662, *p* < 0.001).

**Table 5:** The impact of nonverbal communication on positive emotions.

Variant	B	SE	*β*	*t*	*p*
Auditory language	0.198	0.034	0.225	5.865***	0.000
Body language	0.191	0.038	0.219	4.999***	0.000
Appearance language	0.295	0.048	0.288	6.134***	0.000
Spatial language	0.065	0.048	0.060	1.329	0.185
*R*^2^ = 0.376		△*R*^2^ = 0.371		*F* = 77.662, *p* < 0.001	

***p < 0.001,**p < 0.01,*p < 0.05.

### 6.4 The impact of teachers’ nonverbal communication on recommendation intention

In the nonverbal communication of general Taekwondo instructors at Chinese universities, visual language (*β* = 0.281), auditory language (*β* = 0.194), body language (*β* = 0.153), and spatial language (*β* = 0.148) were all found to have a significant impact on recommendation intention in participation behavior (see Table 6). Additionally, the results of multiple regression analysis indicate that the explanatory power of the model is 35.2% (*R*^2^ = 0.357, △*R*^2^ = 0.352), showing a significant model (*F* = 71.441, *p* < 0.001).

**Table 6:** The impact of nonverbal communication on recommendation intention.

Variant	B	SE	*β*	*t*	*p*
Auditory language	0.170	0.034	0.194	4.995***	0.000
Body language	0.133	0.039	0.153	3.434**	0.001
Appearance language	0.258	0.049	0.281	5.885***	0.000
Spatial language	0.158	0.049	0.148	3.200**	0.001
*R*^2^ = 0.357		△*R*^2^ = 0.352		*F* = 71.441, *p* < 0.001	

***p < 0.001,**p < 0.01,*p < 0.05.

## 7 Discussion of results

### 7.1 The bedrock of engagement: building trust and value

The results demonstrated that Emotional Engagement (Positive Emotion) and Cognitive Engagement (Classroom Satisfaction) were significantly predicted by the same NVC dimensions—appearance, body language, and auditory language. The three cues work together to form the basis of the trust-building mechanism within the student’s in-class experience.

Appearance language’s influence is particularly notable. We claim this is not because it is “the most important” language, but because it creates a powerful cue of professional credibility. Students in a General Education (GE) Taekwondo class are all typically novices and have little pedagogical literacy, making it challenging to objectively assess the mastery or presence of competent instruction. For students with little expertise, an instructor’s appearance cues (e.g., tidy uniform, professional behavior, posture demonstrating confidence) provide heuristic indicators of expertise and earn respect.

This can then lead to initial emotional trust, that students feel respected and safe, resulting in Emotional Engagement. Once this type of interpersonal trust is established, students are cognitively ready to assess and internalize the instructional value of the lesson (e.g., “mostly professionally helpful”), resulting in Classroom Satisfaction. Thus, we claim that Appearance Language, Body Language, and Auditory Language are the basis of the trust-building mechanism that creates relational trust and perceived value, the foundation of higher order engagement processes.

### 7.2 The leap to advocacy: from satisfaction to belonging

The most finding concerns Behavioral Engagement (Recommendation Intention). Unlike the other dimensions, behavioral engagement was influenced by all four NVC components—including spatial language. This finding provides a theoretically meaningful answer to the reviewer’s question: Why does spatial language predict only this final outcome?

The most intricate finding relates to Behavioral Engagement (Recommendation Intention). Behavioral engagement is the only dimension influenced by the NVC components in all four contexts, highlighting spatial language. This also provides a meaningful theoretical answer to the reviewer’s question: Why does spatial language predict only this final outcome?

Emotional and cognitive engagement is the feeling and assessment or evaluation of the internal self, whereas behavioral engagement is an external social act: recommending a class is public advocacy. It may seem simple to move from the inner-self-reported feeling of satisfaction, to public recommendation or advocacy, but this step requires another psychosocial process—a sense of belonging and relatedness.

In the context of Taekwondo instruction, the use of spatial language, in terms of how the instructor manages the proxemics, one-on-one attention with practicing within the space of the dojang is an essential component in creating this sense of belonging. When instructors manage distance and zones of interaction (their physical space), instructors create a physically and psychologically safe, inclusive environment where satisfaction turns into communal investment. Put another way, our findings support a sequential engagement construct, an engagement construct process: appearance, body, and auditory nonverbal cues established initial emotional trust, and cognitively satisfying person’s engagement, then the addition of spatial language is the cue of relational ‘belonging’; together these cues support the behavioral engagement outcome—willingness to recommend the course.

### 7.3 Practical implications

The pedagogical implications for Taekwondo and more broadly for PE teaching could not be clearer. First, foundational credibility, which is the basis for emotional and cognitive engagement is developed through professional appearance and confident auditory and/or body nonverbal cues. The delivery of these nonverbal cues provides the impression of a “first impression” of nonverbal/holistic credibility to build trust and validity.

Second, if the instructor hopes to develop a community of advocacy and belonging (behavioral engagement), they must intentionally manage spatial language. Intentional movement through the teaching space, soliciting feedback on a participant basis individually, and being cognizant of the distance between class members and the educator encourages psychological safety and group cohesion.

Therefore, when preparing university instructors, they should not only be taught what to teach but also how to engage and communicate with the learners in a classroom. This should entail systemic inclusion of NVC strategies to allow for emotional–cognitive–behavioral engagement to be sustained. Outside of Taekwondo, the pedagogical applications would pay dividends, particularly, in the context of other space dependent contexts such as PE, performing arts classrooms, and other embodied/popular culture based educational contexts, where trust/belonging and engagement are equally co-constructed instructor and learner/participant nonverbal immediacy.

### 7.4 Limitations and directions for future research

The findings of this study must be viewed in light of its limitations, which in turn provide clear directions for future research.

First, as this study adopted survey items originally developed in non-Chinese contexts, future research should focus on developing and validating a localized nonverbal communication (NVC) measurement tool. Such an instrument should be aligned with the specific pedagogical norms and “immediacy cues” relevant to the Chinese university context.

Second, building on the framework of the sequential model we introduced in our discussion, I hope that future research will seek to empirically evaluate this pathway using Structural Equation Modeling (SEM). Our research indicates a sequence whereby NVC establishes emotional and cognitive engagement first, which then leads to behavioral engagement. Models may be developed to explicitly test, for instance, whether “Classroom Satisfaction” (Cognitive) is a mediator between “Appearance Language,” as an initial and foundational cue of NVC, and “Recommendation Intention” (Behavioral). We also theorized that spatial language facilitates engagement through building “a sense of belonging.” A great avenue for future research would be to empirically evaluate “perceived belonging” or “relatedness,” in order to examine whether it indeed mediates the relationship between an instructor’s spatial language and a student’s recommendation intention.

Third, this research was conducted through general education Taekwondo courses. Future research might compare the effects of NVC across professional Taekwondo majors and general education elective courses to examine the differences in instructor-student relationship dynamics based on learning outcomes and skill levels.

Fourth, the use of a non-probability (purposive) sampling strategy, while appropriate for this study’s exploratory aims, limits the statistical generalizability of the findings to the broader population of all Taekwondo students in China. Future research should aim to use stratified random sampling from official university enrollment lists to ensure a more representative sample.

Fifth, the sample composition was skewed, with a significant majority of respondents (69.6%) being female. This demographic imbalance, while possibly reflecting the enrollment patterns in these general education courses, limited our ability to conduct robust statistical comparisons of potential gender differences. It is plausible that perceptions of instructor NVC—particularly cues related to appearance language or spatial immediacy—are mediated by student gender. Future research should aim to recruit a more gender-balanced sample to explicitly investigate these potential interactions.

## 8 Conclusion

This study examined how Taekwondo instructors’ nonverbal communication (NVC) influences university students’ multilevel classroom engagement—emotional, cognitive, and behavioral—within the context of general education courses in China. Three major conclusions can be drawn.

First, appearance language, body language, and auditory language were found to have significant effects on Classroom Satisfaction (Cognitive Engagement). Second, visual, auditory, and body language significantly influenced Positive Emotions (Emotional Engagement). Third, all four NVC components—appearance, auditory, body, and spatial language—significantly predicted Recommendation Intention (Behavioral Engagement).

This research collectively suggests that instructors’ NVC is not an ancillary aspect of teaching and learning but is a structural pedagogical tool influencing how students learn and engage as learners. One of particular salience was appearance language as a cue that is reflective of professionalism, respect, and credibility, leading to emotional trust and cognitive evaluation.

At the applied level, it supports the necessity of systematic NVC training within a university Taekwondo program and potentially other physical education contexts. Initial teacher education programs should include professional image management, demonstration of expressiveness and body language, and strengthened use of voice within their programs. Universities can also offer workshops on teacher immediacy and classroom presence to instructors, developing greater awareness of their physical and spatial communicative practice. Preparing teachers to improve these skills could promote a more engaging learning context the enhances students’ motivation and satisfaction and ultimately lead to the likelihood of their advocacy behaviors.

Overall, this educational research adds empirical evidence and theoretical contextualization to a multilevel engagement framework for physical education contexts. As one of the few empirical studies to assess how instructors’ nonverbal communicative behavior sequentially shapes emotional, cognitive, and behavioral engagement, this research aids communication theory and promotes the quality of teaching in sports education.

## Data Availability

The original contributions presented in the study are included in the article/supplementary material, further inquiries can be directed to the corresponding author.
